# Tomato Potato Psyllid *Bactericera cockerelli* (Hemiptera: Triozidae) in Australia: Incursion, Potential Impact and Opportunities for Biological Control

**DOI:** 10.3390/insects14030263

**Published:** 2023-03-07

**Authors:** Shovon Chandra Sarkar, Séverin Hatt, Andrew Philips, Mahjuba Akter, Stephen Paul Milroy, Wei Xu

**Affiliations:** 1 Food Futures Institute, Murdoch University, Murdoch, WA 6150, Australia; 2 Agroecology and Organic Farming, Institute of Crop Science and Resource Conservation (INRES), University of Bonn, 53121 Bonn, Germany

**Keywords:** invasive species, solanaceae, natural enemy, biological control, conservation biological control

## Abstract

**Simple Summary:**

The tomato potato psyllid, *Bactericera cockerelli,* is an important insect pest of potato, tomato, and other solanaceous crops. *B. cockerelli* was first detected on mainland Australia in Perth, Western Australia, in early 2017, which poses a major threat to Australian vegetable industries. It damages directly by feeding and indirectly as a vector of *Candidatus* Liberibacter solanaceaerum (CLso) that causes zebra chip disease in potatoes. Thus far, *B. cockerelli* has not been reported in other states of Australia. As *B. cockerelli* has been reported resistant to insecticides in USA and Mexico, biological control with native natural enemies may provide a viable alternative strategy to manage the insect. In this review, we begin with an overview of information on *B. cockerelli*, its incursion into Australia, and its impact on crops and current management. Opportunities to develop biological control strategies to alleviate the dependence on synthetic insecticides are also considered in this review. Moreover, the potential of existing natural enemies to contribute toward regulating populations of *B. cockerelli* in the field and the challenges ahead to strengthen the key role they can play through conservation biological control are also discussed.

**Abstract:**

Incursion and establishment of an exotic pest may threaten natural habitats and disrupt ecosystems. On the other hand, resident natural enemies may play an important role in invasive pest control. *Bactericera cockerelli*, commonly known as the tomato-potato psyllid, is an exotic pest, first detected on mainland Australia in Perth, Western Australia, in early 2017. *B. cockerelli* causes direct damage to crops by feeding and indirectly by acting as the vector of the pathogen that causes zebra chip disease in potatoes, although the latter is not present in mainland Australia. At present, Australian growers rely on the frequent use of insecticides to control *B. cockerelli,* which may lead to a series of negative economic and environmental consequences. The incursion of *B. cockerelli* also provides a unique opportunity to develop a conservation biological control strategy through strategically targeting existing natural enemy communities. In this review, we consider opportunities to develop biological control strategies for *B. cockerelli* to alleviate the dependence on synthetic insecticides. We highlight the potential of existing natural enemies to contribute toward regulating populations of *B. cockerelli* in the field and discuss the challenges ahead to strengthen the key role they can play through conservation biological control.

## 1. Introduction

The tomato potato psyllid, *Bactericera cockerelli* (Šulc) (Hemiptera: Triozidae), is an invasive polyphagous pest that is spreading globally both naturally and via human activity that damages a wide variety of crops, especially in the Solanaceae [1,2]. In February 2017, *B. cockerelli* was first detected in mainland Australia in Perth, Western Australia (WA), and since then, its range has expanded rapidly over the southwest of the state [3]. Thus far, *B. cockerelli* has not been reported in other states of mainland Australia, but if this spread occurs, it may cause substantial disruption to solanaceous crops and huge economic losses in the Australian vegetable industry.

Previously, Butler and Trumble [4] reviewed the biology and ecology of *B. cockerelli* and presented a set of management strategies to control *B. cockerelli* and the pathogen it transmits. More recently, Olaniyan et al. [5] updated the current knowledge on *B. cockerelli* and the control strategies in the context of an imminent risk of invasion in China. Similarly, Vereijssen et al. [6] and Vereijssen [7] did so in the context of New Zealand, where *B. cockerelli* has become established. In the present review, the current situation of *B. cockerelli* in Australia is described, and its potential spread across the country is discussed in light of its biological properties and within this country’s ecological context. Finally, the role of native and resident non-native species of natural enemies and host plant species of *B. cockerelli* for conservation biological control are also discussed.

To date, the application of insecticides has been the main way to control *B. cockerelli* worldwide; repeated applications are often involved (as reviewed by Butler and Trumble [4]). In the present review, the reliance on chemical control is discussed by highlighting the detrimental ecological effects of insecticide use against *B. cockerelli* in Australia. As an alternative, opportunities for biological control are presented by reviewing the current challenges for its development and implementation in Australia. Notably, a diversity of potential host plants and natural enemies of *B. cockerelli* in Australia is identified, and strategies for biological control through habitat management to conserve them in Australian agricultural landscapes are suggested. Our aim is to provide information towards the development of more ecologically based and environmentally friendly strategies for the management of *B. cockerelli* using existing natural enemies in Australia.

## 2. *B. cockerelli* as a Crop Pest

*B. cockerelli* is an economically important agricultural insect pest for a range of crops. Its host range encompasses over 40 species of solanaceous plants and can utilize species from over 20 families [8,9,10,11].

The sum of biological characteristics of *B. cockerelli*, including its small size (the adult body can vary from 2.5 to 2.75 mm), short developmental times (12–44 days with an average of 15.4 days), high reproductive rate (on average, up to 330 eggs over a female lifetime), flying skill and its superior host acquisition capability contributes to make it an invasive pest [4,9,10,12]. Invasive pests often exhibit high phenotypic plasticity, which makes them difficult to differentiate from native species of the same family at the early stages of development [13,14]. *B. cockerelli* goes through three stages of development: egg, nymph, and adult. Eggs of *B. cockerelli* are mainly deposited near the leaf edge and on the lower leaf surface but may be deposited on any part of the plant, although this varies with the host plant [15]. Nymphs of *B. cockerelli* are usually found in shaded locations (mostly on the lower leaf surfaces) and usually remain sedentary during all five nymphal stages [9]. The adults are pale green in color when they emerge darker in color within 2–3 days and later can become grey or black [16]. Adults have well-developed wings and legs, are good fliers, and jump readily when disturbed [1,17,18]. The optimum temperature for reproduction and development is 24–27 °C, while temperatures above 31 °C may cause death [19,20]. Henne et al. [21] reported that both *B. cockerelli* nymphs and adults are cold tolerant: nymphs survived at −15 °C for 24 h, and 50% of adults survived at −10 °C for 24 h. However, the development and survival of *B. cockerelli* are impacted by the identity of the host plant [22].

*B. cockerelli* can cause severe economic losses by damaging host plants through phloem-feeding and indirectly by acting as a vector of the alphaproteobacterium *Candidatus* Liberibacter solanacearum (CLso), a bacterial pathogen that can reduce crop yield significantly [5]. In the absence of a pathogen, feeding by *B. cockerelli* induces a host disorder known as “psyllid yellows” disease. Psyllid yellows are characterized by foliar symptoms, including chlorosis, upward rolling of younger leaves, stunting, and, in severe cases, may result in early plant death [23,24]. For potatoes, tuber development can be greatly affected, resulting in misshapen tubers and abnormal sprouting [24]. Tomatoes also exhibited significant yield reduction because of this disease. Fruit set, size, yield, and quality (including shape and texture) decrease significantly [22,24]. In extreme cases, total yield loss can occur [10,25,26]. Through the transmission of the CLso, *B. cockerelli* attacks can cause “zebra chip” disease [27,28]. Potatoes affected by zebra chip exhibit symptoms similar to psyllid yellows [28]. The name “zebra chip” comes from the dark lines that appear when infected tubers are fried, which makes fresh and processed potatoes unusable for the market [29].

The zebra chip disorder can spread rapidly within a production region of Solanaceous crops [30,31]. Therefore, the economic impact of zebra chip disease has been severe on the worldwide export business of Solanaceae [32,33]. After the detection of *B. cockerelli* in WA, the export of all live plant material, not only potato tubers but also all horticultural produce potato tubers market access from WA to other states of Australia was immediately ceased. The movement of material was reinstated on an industry-by-industry basis. To remove the trade barrier on WA potatoes, an extensive surveillance program for CLso detection began in October 2018 with networks of psyllid traps established in various locations where the psyllid had been identified. To date, the CLso pathogen has not been found in WA. However, in 2018, the disease was first reported in Norfolk Island, an external territory of Australia. The disease was in tomato plants with yellowing symptoms [34].

## 3. Invasiveness and Distribution of *B. cockerelli*

Over the last two decades, *B. cockerelli* has received increasing attention and has been recognized as an important pest of solanaceous crops, especially when it was identified as the key vector of CLso [2,35].

In WA, the state Department of Primary Industries and Regional Development (DPIRD) identified *B. cockerelli* in Perth in February 2017 and found that it was already widespread around the Perth metropolitan area [3]. Olaniyan et al. [5] theorized that *B. cockerelli* may have arrived in Australia either through legal importation or through the smuggling of infested host plant material. Previously, Biosecurity Australia [36] and Plant Health Australia [37] mentioned that international trade of fresh fruits, potato tubers, nursery stocks, or hitchhiker transportation of psyllids on plant or non-plant materials were possible means of entry to Australia.

In Oceania, *B. cockerelli* was first found in New Zealand in 2006 [38], and since then, it has established populations all over that country, where it causes extensive damage to crops in the Solanaceae [33]. It is not clear how *B. cockerelli* arrived in New Zealand. Still, it is believed that, through international trade or through smuggled primary host plant materials, it was introduced from the North American region between 2000 and 2006 [25,32,33,34,39]. After the discovery of *B. cockerelli* in New Zealand, the Australian government implemented strict measures to prevent the invasion of this pest to Australia [40]. To date, four haplotypes (western, central, northwestern, and southwestern) of *B. cockerelli* have been identified worldwide. The haplotype found in WA is the western haplotype, previously found in Norfolk Island, New Zealand, Honduras, Guatemala, Mexico, and the western USA [39,41].

Nonetheless, *B. cockerelli* has so far only been observed in WA in mainland Australia. There are several possible reasons why *B. cockerelli* has not been discovered in other Australian states so far. The most likely reason is that there is potentially a large host gap between the agricultural areas of WA and those of the other states of Australia. From the edge of the wheat-sheep zone east of Esperance (WA) to the areas west of Ceduna (South Australia) is a distance of some 1000 km. Between is a narrow coastal strip with low rainfall (200–300 mm), strongly seasonal, bordering the desert areas of central Australia. However, the higher rainfall areas where outdoor horticulture is established are much more widely separated than the cereal production areas. Because the potential of Australian native species to host *B. cockerelli* is poorly known [40], the significance of this geographical separation is unclear. While there is the potential for *B. cockerelli* to move from WA to the other states via commercial shipments of products, inter-state, and intra-state quarantine regulations are in place. Secondly, the incursion and establishment of *B. cockerelli* may be hampered because of competition with native psyllid and other insect species. Liu and Trumble [22] observed competition between native and invasive psyllid species in North America. However, there is very little information available about potential interactions of *B. cockerelli* and Australian native psyllids or other insects that may occupy similar ecological niches.

It should also be recognized that at the early stage of incursion, the density of *B. cockerelli* will be low, and their smaller size and cryptic behavior create difficulties in identification. Therefore, at the early stage of incursion, *B. cockerelli* may remain unnoticed in the extensive agricultural landscape of Australia. However, Australia’s eastern and southern states are located in the optimal climate regions for *B. cockerelli* and thus should be recognized as being at high risk [41].

## 4. Chemical Control of *B. cockerelli* and Ecological Effects

Growers have mainly relied on the frequent application of insecticides to control *B. cockerelli* worldwide, which has also been the basis for managing zebra chips as there is no known treatment available for CLso [42,43].

Insecticides such as cyantraniliprole, spinetoram, oxamyl, imidacloprid, spirotetramat, and tolfenpyrad have been tested under greenhouse conditions against *B. cockerelli* adults and nymphs in the Columbia Basin [44]. The results showed that spinetoram and high rates of cyantraniliprole had the greatest immediate and residual activity against *B. cockerelli* adults. Cyantraniliprole and oxamyl were effective against nymphs, with no differences between rates [45]. The effects of different chemical insecticides may vary when applied to different plants or cultivars [46,47]. 

The management of *B. cockerelli* is difficult due to their cryptic habits, as the nymph of *B. cockerelli* usually lives on the lower side of the leaf, which reduces the impact of contact insecticides. Moreover, repeated chemical treatments are frequently necessary to ensure ongoing control of the pest. For example, in Mexico, potato growers have been required to apply up to 30 foliar sprays per season [48]. In New Zealand, a grower can reach nearly fifteen sprays per season [32]. Such high usage of synthetic insecticides can potentially lead to a series of adverse ecological effects, including insecticide resistance in *B. cockerelli*, residue problems, environmental contamination, toxicity to beneficial and non-target organisms, species displacement, and disruption of biological control [49,50,51,52]. In addition, chemical control of *B. cockerelli* is costly (around US $700 per hectare) [43,53]. Control measures may seriously erode profit margins when high numbers of applications are needed. Despite this, the reduction of *B. cockerelli* numbers in potato fields may not decrease CLso infestation [54], and thus saleability of the product is still reduced. Moreover, the application of chemical insecticides can lead to secondary pest outbreaks [55]. For example, pyrethroid insecticides can induce higher oviposition by *B. cockerelli* females and also flare other pests such as aphids (Hemiptera: Aphididae) and mites (Acari: Tetranychidae) [56].

A study of insecticides, including abamectin, cyantraniliprole, pymetrozine, flonicamid, and spirotetramat on *B. cockerelli* in WA revealed that abamectin, cyantraniliprole, and spirotetramat effectively suppressed *B. cockerelli* in pepper, tomato, and potato [3]. Of the feeding deterrents, flonicamid showed some degree of *B. cockerelli* suppression, while pymetrozine did not provide any suppression of *B. cockerelli* [3]. These two compounds have also shown limited impact in reports from the USA (Liu and Trumble 2005). To date, there has been no report of insecticide resistance in *B. cockerelli* in Oceania [5]. In California, Liu and Trumble [22] observed *B. cockerelli* resistance to imidacloprid (LC_50_ for 50% of *B. cockerelli* nymphs). Prager et al. [54] also reported resistance of *B. cockerelli* to imidacloprid in South Texas (USA). Thus, while there is not yet evidence of insecticide resistance in Australia, in light of the reports from the USA and Mexico [51,57], it can be expected that the *B. cockerelli* population of Australia and New Zealand will eventually show resistance against chemical insecticides unless resistance management strategies are developed and deployed across industries. However, this relies on (i) the availability and registration of effective insecticides with a range of modes of action and (ii) the level of industry coordination and compliance with resistance management strategies. Resistance has become a major concern, and synthetic insecticides may not be a long-term solution for *B. cockerelli* management [5].

## 5. Prospects for Biological Control Using Existing Natural Enemies in Australia

There appear to be significant opportunities to use natural arthropod enemies for the management of *B. cockerelli* in Australia as an alternative strategy to insecticide application or as part of an integrated management approach. This may also contribute to the development of resistance management strategies. A number of generalist predator species in Australia may have the potential as biocontrol agents for this new invasive pest (Table 1). Generalist predators are more resilient to pest invasions and have a higher likelihood of being able to utilize a new species as prey; thus, they can often play an important role in developing biological control strategies against new invasive pest species [58,59,60,61,62,63,64,65].

Research over the past two decades has demonstrated the potential of several species to utilize *B. cockerelli* as prey and, in some cases, has indicated the potential to control populations. Among the Coleoptera, the 11-spotted ladybird beetle, *Coccinella undecimpunctata* L. (Coleoptera: Coccinellidae), and the large spotted ladybird beetle, *Harmonia conformis* Boisduval (Coleoptera: Coccinellidae) can prey upon all life stages of *B. cockerelli,* and they were identified by McDonald et al. [110] as potential biocontrol agents for potato in New Zealand. Convergent ladybird beetle *Hippodamia convergens* Guérin-Méneville (Coleoptera: Coccinellidae) has been reported as a predator of *B. cockerelli* in California [42]). Laboratory and glasshouse experiments indicated the Southern ladybird beetle, *Cleobora mellyi* Mulsant (Coleoptera: Coccinellidae), as a potential biocontrol agent for *B. cockerelli* in New Zealand [111]. This ladybird species, imported to New Zealand from Australia, can consume up to 100 nymphs of *B. cockerelli* in 24 h [112].

Among the Neuroptera, the brown lacewing *Micromus tasmaniae* Walker (Neuroptera: Hemerobiidae) can prey upon all life stages of *B. cockerelli,* and it was identified by MacDonald et al. [110] as a potential biocontrol agent for potato in New Zealand. It is found widely in potato fields in New Zealand and is considered a dominant predator. Importantly, it was observed to attack *B. cockerelli* early in the potato production cycle [113]. Both *M. tasmaniae* and *Melanostoma fasciatum* Macquart (Diptera: Syrphidae) are reported to be able to consume more than 12 nymphs in 24 h [110].

In the Americas, various Hemiptera have been found to manage *B.cockerelli*. In Mexico, Pineda et al. [114] identified the predatory mirid, *Engytatus varians* Distant (Hemiptera: Miridae), as a promising biocontrol agent of *B. cockerelli* on tomatoes. However, *E. varians* can also damage tomato plants [114]. In Mexico, Perez-Aguilar et al. [115] were able to control *B. cockerelli* without crop damage on greenhouse tomatoes. When the predatory mirid, *Dicyphus hesperus* Knight (Hemiptera: Miridae), was evaluated as a biocontrol agent of *B. cockerelli* and *Bemisia tabaci* Gennadius (Hemiptera: Aleyrodidaeon) in greenhouse tomatoes, Calvo et al. [116] observed that it could significantly reduce the numbers of both pests. It has been known for some time that the minute pirate bug, *Orius tristicolor* White (Hemiptera: Anthocoridae), can consume a considerable number of *B. cockerelli* nymphs and adults in solanaceous crops [4,117]. Similarly, Butler and Trumble [42] confirmed the western big-eyed bug, *Geocoris pallens* Stål (Hemiptera: Geocoridae), to be a predator of *B. cockerelli* in potato. This species had been previously noted by Pletsch [23] in the northwestern region of the United States.

In addition to predators, parasitoids might also have potential as biological control agents. *Tamarixia triozae* Burks (Hymenoptera: Eulophidae), a parasitoid of the Asian citrus psyllid (*Diaphorina citri* Kuwayama), has between 60% and 80% parasitism rate on *B. cockerelli* [118]. However, Luna-Cruz et al. [119,120] and Liu et al. [121] reported the high mortality of adult *T. triozae* due to its susceptibility to insecticides, emphasizing the challenge of combining insecticide usage and biological control in IPM [50,122,123].

Although predators from a range of genera and families are known to attack *B. cockerelli*, whether measured in the field, glasshouse, or laboratory, substantially more research is needed to establish which species are likely to be viable biological control agents. Key questions relate to the capacity to establish and maintain useful predator populations in the field and the likely choice of prey by polyphagous predators.

Of primary importance will be assessing whether a species can survive and establish useful populations in the crop environment in the geographical location of interest and at the time of year when suppression of the pest population is likely to be needed. This requires autecological data for both the pest and the predator across the likely range of deployment. In Australia, this will be challenging given the diversity of climatic zones and agroecological systems in which solanaceous crops are produced.

Butler and Trumble [42] have questioned whether biological control is feasible given the high reproductive rate of *B. cockerelli*. This will depend not only on the voracity of the predator but also on the timing of its release relative to the likely development of the *B. cockerelli* population and on the predator’s own reproduction rate [110]. In New Zealand, modeling capacity has been developed to predict the timing of likely *B. cockerelli* pressure based on the temperature conditions at the beginning of the season [124]. Whether this can be usefully adapted to Australian production systems will depend on the size of overwintering pest populations, their likely movement into crops, and the applicability of the model assumptions to Australian production environments.

The capacity of the predator population to remain in the system after release, particularly after *B. cockerelli* numbers have been suppressed, will influence release strategies. If the predator population declines too far, it may not have the capacity to respond to a new influx of the pest, and populations may need to be supplemented. Here again, polyphagous predators may present an advantage given their ability to utilize a range of food sources. If alternative food sources are present when the target pest declines, it will increase the likelihood of the predator remaining in the location. The possibility of conservation biological control to assist with maintaining predators in the environment will be discussed in the next section.

The species presented in Table 1 are already resident in Australian agroecosystems. This reflects their capacity to establish and maintain populations in the environment and makes them logical choices for evaluation. However, whether the population size is large enough or can build up quickly enough to provide a meaningful impact on *B. cockerelli* pest populations needs to be evaluated and appropriate intervention thresholds established. An interesting aspect in this regard will be the effect of utilizing *B. cockerelli* as a major food source (in contrast to established food sources) on the life expectancy and reproductive rate of the predator.

In many situations [125,126,127], perhaps more likely in field-grown crops than in the greenhouse, questions of prey choice and prey switching may also be significant. If alternative food sources are present, is the predator likely to utilize *B. cockerelli*, the alternative food source, or both? Calvo et al. [116] observed that *D. hesperus* established, reproduced, and significantly controlled two major Solanaceae pests, *B. tabaci* and *B. cockerelli* at the same time in the greenhouse. It is possible that this may be influenced by the previous feeding experience of the predator, whether this was under conditions of artificial rearing or in the field. That is, is it possible that a predator will adapt more quickly to the utilization of *B. cockerelli* if the species was part of its diet in the past?

There has been a recent trend towards using multiple natural enemies for the management of *B. cockerelli* to get more stability in pest control. de Lourdes Ramírez-Ahuja et al. [128] observed that the simultaneous release of the parasitoid *T. triozae* and the predatory mirid *D. hesperus* have an additive effect on the reduction of *B. cockerelli* populations. Entomopathogenic fungus *Beauveria bassiana* and the parasitoid *T. triozae* have also displayed potential for wide applications for controlling *B. cockerelli* worldwide [118,121,129], and Tamayo-Mejía et al. [130] suggested their combined application for biological control of *B. cockerelli.* However, the sub-lethal effects of *B. bassiana* on *T. triozae* would reduce their efficiency in *B. cockerelli* control [130,131]. These undesirable effects can possibly be minimized by the synchronized application of the two agents [131]. Additionally, resource competition might occur between predator species when they compete for the same prey [132,133,134]. Therefore, careful planning and management are required for combinations of biocontrol agents to control *B. cockerelli.*

**Figure 1:**
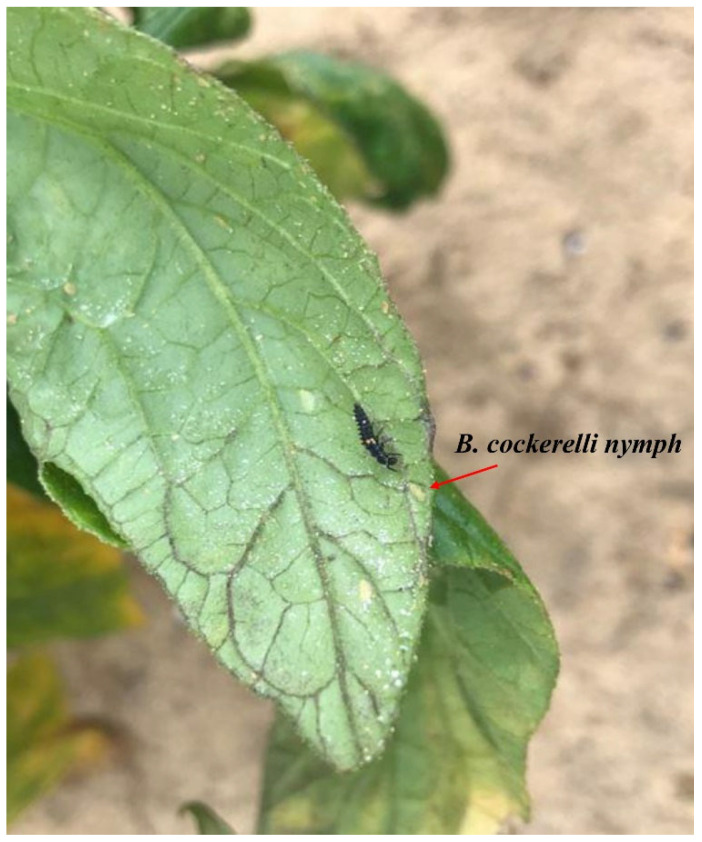
*Coccinella transversalis* larvae praying on *Bactericera cockerelli* nymphs on capsicum in the field.

From our preliminary field survey, laboratory, and glasshouse study (unpublished data), Australian native and non-native resident predators can play an important role in the biocontrol of *B. cockerelli*. Ladybird beetles (*H. conformis*, *Cheilomenes sexmaculata* Fabricius (Coleoptera: Coccinellidae), *Coccinella transversalis* Fabricius (Coleoptera: Coccinellidae) and *Hippodamia variegata* Goeze (Coleoptera: Coccinellidae)) and lacewings (*Mallada* sp. Schneider (Neuroptera: Chrysopidae) and *M. tasmaniae*) have been observed feeding on *B. cockerelli* in capsicum fields (Figure 1). Further, non-native resident species *H. variegata* and native species *C. transversalis* ladybird beetles successfully survived and suppressed *B. cockerelli* populations in the laboratory and in cage experiments using glasshouse-grown tomato plants [135,136]. Suppression by the ladybird beetles resulted in a positive influence on plant chlorophyll content and biomass.

## 6. Conservation of *B. cockerelli* Natural Enemies in Agricultural Landscapes

Conservation biological control is the practice of preserving the habitat surrounding fields to allow the natural enemies of pests to maintain a constant presence in the ecosystem, thus allowing faster colonization of the crops [137]. Faster colonization, in turn, would lead to greater and more timely control of pests. While a diversity of potential *B. cockerelli* predators and parasitoids has been identified (Table 1; see also the previous section), we have conducted a field survey to identify the effective natural enemies of *B. cockerelli* occurring in the environment in Western Australia (unpublished data). To our knowledge, habitat management to enhance biological control against this pest has not been evaluated either in Australia or elsewhere.

Research into conservation biological control of other pests in Australia and into ecological services more generally may guide research aiming at testing habitat management strategies for the control of *B. cockerelli*. Some previous results may prove directly applicable, such as information on population dynamics or movement of generalist predators in the environment where these species have also been identified as predators of *B. cockerelli*. In Australia, non-crop habitats such as native forests and remnant woodlands are known to host predators, and parasitoids of crop pests benefit from woody shelterbelts and windbreak hedgerows, as well as remnant native grasslands (reviewed by Gagic et al. [138]). Whenever possible in Australia, native plant species should be chosen when designing and implementing semi-natural habitats since it has been shown that native species host more natural enemies and fewer pests in comparison to exotic plant species [139].

The proportion of native vegetation and its fragmentation and spatial distribution varies dramatically across the agricultural areas of WA (https://dwer.wa.gov.au/ (accessed on 1 May 2022)). In the central wheat belt particularly, the proportion of native vegetation is low, and the remnants are fragmented. By contrast, in the higher rainfall zones south of Perth, that is, west of approximately 116°30′ E, there are more extensive areas of forest and reserve (Geographical Information Services 2016). It is in the higher rainfall areas of WA that *B. cockerelli* is currently found and where the main crop hosts, potato, tomato, capsicum, and eggplant, are produced. In some cases, tomato and potato crops are grown adjacent to forests or remnant native vegetation. The value of this contact needs to be assessed in terms of the colonization of crops by predators. However, it should also be noted that WA has a very high diversity of native species of Solanaceae. The capacity of these species to host *B. cockerelli* has not been explored. Thus, proximity to native vegetation may present risks and benefits that must be evaluated.

Flower-rich semi-natural habitats have also been explored to bolster populations of beneficial species. Sowing strips of buckwheat (*Fagopyrum esculentum* Moench, Polygonaceae) and coriander (*Coriandrum sativum* L. Apiaceae) is possible adjacent to fields of Solanaceae [140,141,142]. These flowers are known to be visited by several natural enemies (Coccinellidae, Chrysopidae, Syrphidae, parasitoids) of *B. cockerelli* and enhance fitness by providing alternative foods [143,144]. Crops of species such as lucerne (*Medicago sativa* L., Fabaceae) in spring and sorghum (*Sorghum bicolor* L. Moench, Poaceae) in summer, can host a high abundance of generalist predators, including *Coccinella* spp., *Nabis* spp., and *Micromus* spp. [145]. In their review, Rizvi et al. [146] highlighted the potential role of sorghum (*Sorghum bicolor* L. Moench, Poaceae) as a banker plant sown at the border of capsicum fields and indicated that the technique is now being trialed by growers in WA.

Successful experiences of habitat management conducted outside Australia enhancing natural enemies of *B. cockerelli* could also contribute to the development of effective conservation biological control against this pest. Many of the *B. cockerelli* natural enemies present in Australia (Table 1) depend on nectar and pollen for surviving and reproduce, i.e., adult Syrphidae, Eulophidae, Encyrtidae [118,147], and adult Coccinellidae, *Chrysoperla* spp. and *Orius* spp. are mixed feeders [148,149]. Notably, flower-rich habitats could use multi-species and functional mixtures to attract and support a diversity of natural enemies throughout the season [150]. In Switzerland, for instance, wildflower strips composed of a mixture of annual forb species significantly enhanced the abundance of adult lacewings and eggs and the richness of predatory hoverflies in adjacent potato crops [151]. In China, annual wildflower strips sown outside but along greenhouses enhanced the abundance of *Orius* sp. and lacewings in eggplants (*Solanum melongena* L., Solanaceae) cultivated indoors [152]. In the USA, hedgerows composed of native shrubs and bordered by native grasses enhanced the abundance of predatory ladybird beetles, including *Hippodamia* spp., in adjacent tomato fields [153].

The management of native vegetation, the utilization of existing crops in the landscape as hosts, and the introduction of planted strips of ‘banker plants’ all present possibilities for enhancing the biological control of *B. cockerelli*. However, their benefit and strategies for their implementation will need to be considered across the diversity of the Australian production environments and systems. Where these approaches contribute to enhancing populations of generalist predators, it is possible that they may enhance the management of a range of pest species and so have a wider benefit.

## 7. Conclusions

*B. cockerelli* is spreading internationally. It poses a threat to the production of globally important Solanaceous crops both directly and as a vector for CLso. The use of chemical insecticides has already caused problems through the development of insecticide resistance in the psyllid and through the disruption of its natural enemies. Research, development, and implementation of biological control against *B. cockerelli* present an important alternative approach to control that may also contribute to the development of resistance management strategies or be incorporated as a possible component of Integrated Pest Management approaches.

The diversity of generalist natural enemies of *B. cockerelli* that are already present in Australia is promising. This shifts the emphasis for future research away from identifying potential predators and towards quantifying their benefit within Australian agroecological systems. Questions of population dynamics within these systems and the influence of the prey species on the development and reproduction of the predators are of primary importance. These factors are likely to vary substantially across the continent. A major variable will be the influence of surrounding vegetation on both the pest and predator species.

## Figures and Tables

**Table 1:** Natural enemies that occur in Australia and that have been identified in other countries as preying upon or parasitizing *Bactericera cockerelli*.

Class	Order	Family	Species	Distribution in Australia	References
Arachnida	Acari	Anystidae	*Anystis baccarum* Linné	NSW, QLD, SA, TAS, VIC	Holm and Wallace [66]
		Phytoseiidae	*Phytoseiinae*	NSW, NT, QLD, SA, TAS, VIC, WA	Walter and Beard [67]; Beard [68]
	Araneae	Araneidea	*Cyclosa* sp.; *Nesoscona* sp.	All states	Whyte and Anderson [69]
		Dictynidae	*Ixeuticus candidus* Koch	All states	New [70]
		Linyphiidae	*Laetesia raveni* Hormiga and Scharff	NSW, QLD, SA	Hormiga and Scharff [71]
		Miturgidae	*Cheiracanthium* spp.	All states	Raven [72]; Hogg et al. [73]
		Oxyopidae	*Oxyopes* spp.	All states	Vink and Sirvid [74]; Baehr et al. [75]
		Salticidae	*Habronattus* spp.	All states	Richardson et al. [76]
		Theridiidae	*Theridion* sp.	All states	Framenau [77]
		Thomisidae	*Thomisidae* spp.	All states	Szymkowiak [78]
Insecta	Coleoptera	Coccinellidae	*Cleobora mellyi* Mulsant; *Hippodamia variegata* Goeze; *Coccinella transversalis* Fabricius, etc.	All states	Pope [79]; Lipiski [80]; Franzmann [81]; Slipinski [82]; Li et al. [83]
		Melyridae	*Phycosecis litoralis* Pascoe	All states	Beutel and Pollock [84]
		Staphylinidae	*Platystethus* sp.	All states	Chandler [85]
	Dermaptera	Forficulidae	*Forficula auricularia* L.	NSW, QLD, SA	Binns et al. [86]
	Diptera	Dolichopodidae	*Asyndetus* Loew; *Chrysotus* spp. *Medetera* spp. Bickel	All states	Bickel [87,88,89]
		Syrphidae	*Allograpta* Osten Sacken; *Sphaerophoria* spp.; *Melanostoma* sp.	All states	Mengual and Thompson [90]; Robertson et al. [91]; Finch and Cook [92]
	Hemiptera	Anthocoridae	*Orius* Wolff; *O.* gracilis sp. n.	NSW, NT, QLD, WA	Postle et al. [93]
		Berytidae	*Rhyparochrominae* spp.; *Berytinus* spp.	NSW, QLD, SA, TAS	Slater and Woodward [94]; Wheeler and Schaefer [95]
		Nabidae	*Nabis kinbergii Reuter*	All states	Ma et al.l. [96]
		Geocoridae	*Germalus* Stål; *Stylogeocoris* Montandon	NT, QLD, SA, VIC	Malipatil and Blackett [97]
		Miridae	*Creontiades* Distant; *Engytatus passionarius* sp. nov.	All states	Malipatil and Cassis [98]; Minghetti et al. [99]
		Pentatomidae	*Oechalia schellenbergii* Guérin	All states	Sands and Coombs [100]
		Reduviidae	*Emesopsis* spp.	All states	Tatarnic et al. [101]
	Hymenoptera	Encyrtidae	*Baeoanusia xanthopleuron* sp. n.; *Avetianella coombsi* sp. n.	NSW, QLD, SA, VIC	Schmidt and Noyes [102]
		Eulophidae	*Tamarixia* spp.	NSW	Zuparko et al. [103]
		Formicidae	*Linepithema humile* Mayr	NSW, QLD, SA, TAS, VIC, WA	Walters and Mackay [104]
	Neuroptera	Chrysopidae	*Chrysoperla* spp.; *Mallada signatus* Schneider; *Lauraya retivenosa* sp.n.	NSW, QLD, SA, TAS, VIC, WA	Smithers [105]; Winterton [106]
		Hemerobiidae	*Micromus tasmaniae* Walker	ACT, NSW, QLD, SA, TAS, VIC, WA	New [107,108]
	Thysanoptera	Aeolothripidae	*Cranothrips* Bagnall; *Cycadothrips* Mound	TAS, VIC	Mound and Marullo [109]

States of Australia: Australian Capital Territory (ACT), New South Wales (NSW), Queensland (QLD), Northern Territory (NT), Western Australia (WA), South Australia (SA), Victoria (VIC), and Tasmania (TAS). Distribution area data collected from ‘Atlas living of Australia (www.ala.org.au).’

## Data Availability

The new data presented in this study are available on request from the corresponding author.
